# Melt layer erosion during ELM-like heat loading on molybdenum as an alternative plasma-facing material

**DOI:** 10.1038/s41598-017-12418-z

**Published:** 2017-09-25

**Authors:** G. Sinclair, J. K. Tripathi, P. K. Diwakar, A. Hassanein

**Affiliations:** Center for Materials Under Extreme Environment (CMUXE), School of Nuclear Engineering, Purdue University, West Lafayette, IN 47907 USA

## Abstract

Transient events that occur during plasma instabilities in fusion reactors impart large heat fluxes onto the surrounding plasma-facing components (PFCs). Erosion and splashing of PFCs can contaminate the plasma and shorten material lifetime. Although tungsten is currently considered the most promising candidate material for future PFCs, concerns over the thermal shock performance during type-I ELMs (transient events expected in fusion devices) necessitate the study of other comparable materials. ELM-like heat loading was applied via a pulsed Nd:YAG millisecond laser on a pristine molybdenum (Mo) surface to measure surface melting and mass loss. One potential advantage of Mo is its higher specific heat of vaporization, which could lead to reduced particle emission. Imaging of the surface after loading revealed that complete surface melting began at 1.0 MJ m^−2^ (heat load parameter of 31.62 MJ m^−2^ s^−1/2^). Photon excitation also increased significantly above 1.0 MJ m^−2^, indicating possible phase change. At 1.4 MJ m^−2^ (44.27 MJ m^−2^ s^−1/2^), *in situ* mass loss measurements found an exponential increase in particle emission, indicating the presence of droplet formation and boiling. Direct comparisons of erosion during pulsed heat loading between PFC candidate materials will ensure that future fusion devices design components with optimal thermal strength.

## Introduction

Instabilities within fusion plasmas compromise the structural integrity of the surrounding plasma facing components (PFCs). Magnetic fusion devices (e.g. ITER) experience periodic discharges of plasma at very high fluxes while operating in the preferred high-confinement mode (H-mode)^1^. Edge-localized modes (ELMs) are events that involve repetitive relaxation of the edge plasma during operation. Different types of ELMs are categorized by their power loss and their peak flux to the divertor region. Type-I ELMs are the most serious, imparting up to 10% of the core plasma energy onto the PFC surface with a repetition rate ranging from 1–10 Hz^2–4^. As a result of intense heating, the PFC surface may crack or melt, leading to component damage and contamination of the fusion plasma^5–7^. Efforts have been made to reduce the magnitude of ELM heat loading through forced relaxation (e.g. pellet injection)^1,8^. However, recent estimates suggest that mitigated ELMs could still possess energy densities up to ~1.0 MJ m^−2^ (at frequencies ~50 Hz)^9–11^. Unmitigated ELMs (giant ELMs) will provide larger heat fluxes on the order of several MJ m^−2^ ^2,12,13^. Determining safe operating windows to minimize cracking and melting, in response to these transient events, will help optimize PFC lifetime and device performance.

Tungsten (W) is currently the leading candidate material for PFCs in current and future fusion devices. The ITER project will use W as the primary divertor material^14^. Advantages of using W in a fusion environment include its high melting point, high thermal conductivity, low sputtering yield, and low tritium retention^15,16^. Both experimental and modeling (TMAP) efforts have successfully characterized the trap energies for different hydrogen isotopes (e.g. deuterium and tritium) in tungsten^17–19^. Unfortunately, poor response of the W surface to low-energy He^+^ irradiation (especially at elevated temperatures) and unmitigated ELMs could present serious problems for its viability as a future PFC. Experiments done in the laboratory and in the Alcator C-Mod tokamak have shown that nanoscale tendrils grow on the W surface in response to low energy He^+^ irradiation at elevated temperatures^20–23^. The nanostructured layer, referred to as “fuzz”, has shown to exhibit reduced thermal, mechanical, and structural properties^15,24,25^. An observed reduction in the thermal conductivity of ~80% could lead to increased levels of erosion due to melting and splashing of the component surface during ELM events^26^.

Recent research shows that molybdenum (Mo) may be a promising PFC alternative^22,23,27–30^. Mo is a high-Z refractory metal with similar advantages to W, including a high melting point, low sputtering yield, and high thermal conductivity^30^. A potential drawback of Mo is its high waste disposal rating under neutron exposure^30^. However, work done on the Tritium Plasma Experiment (TPE) linear plasma device revealed that fractional hydrogen isotope retention could be lower in Mo than in W^31^. Mo also possesses a higher specific heat of vaporization, which will result in less evaporation during transient heat loading^30^. Fuzz formation has been shown to occur on both W and Mo, as well as other refractory metals, for certain fluence regimes and temperature windows^22,23,27,28,32,33^. Recent work has estimated a temperature window for W of 1000–2000 K, and a lower and narrower temperature window for Mo of 823–1073 K^23,27^. Once ITER progresses into the nuclear phase, W surface temperatures will climb into the upper end of the fuzz formation temperature window, making tendril growth a concern^34^. Although the nanostructure remains relatively uncharacterized, the difference in the fuzz formation window for Mo necessitates further analysis of its material response under fusion-relevant conditions.

The presented study investigated surface damage during ELM-like heat loading, which was replicated using long-pulsed laser irradiation. Other laboratory methods for replicating type-I ELM events include the use of plasma accelerators and electron beams^6,35^. The exposures were conducted over a wide range of energy densities to determine how the Mo surface deforms, and eventually melts with increasing intensity. Performing similar types of laser irradiations could be used to drive crystalline phase transitions on Mo oxides to improve semiconductor properties^36^ or induce the nitration of Mo for use in hydrotreatment catalysis of crude oil^37^. Examining the effect of different laser intensities on the surface morphology and potential phase transition of the Mo surface could optimize the different processes previously mentioned. Melting and splashing of the Mo surface during pulsed laser irradiation was measured using a quartz crystal microbalance (QCM), which has previously been used to measure the viscosity of different liquids^38^. *In situ* QCM mass loss measurements provided important insight into the trends in particle emission with increasing energy density. Studying the mechanisms of thermally-induced damage on Mo during transient plasma events will substantively affect the design of future PFCs.

## Results and Discussion

### Changes in surface morphology during pulsed heat loading

Exposing Mo to transient heat loading via long-pulsed laser irradiation provides invaluable information on how a PFC surface is expected to fail at higher intensities. Work done in refs^39,40^ illustrate that material damage is a function of energy density, pulse shape, number of pulses, and base temperature. Therefore, energy density values mentioned below function simply to differentiate between damage regimes, and will change in magnitude under different experimental conditions. Future work will continue to expand on the synergistic relationship between different heat loading parameters.

**Figure 1:**
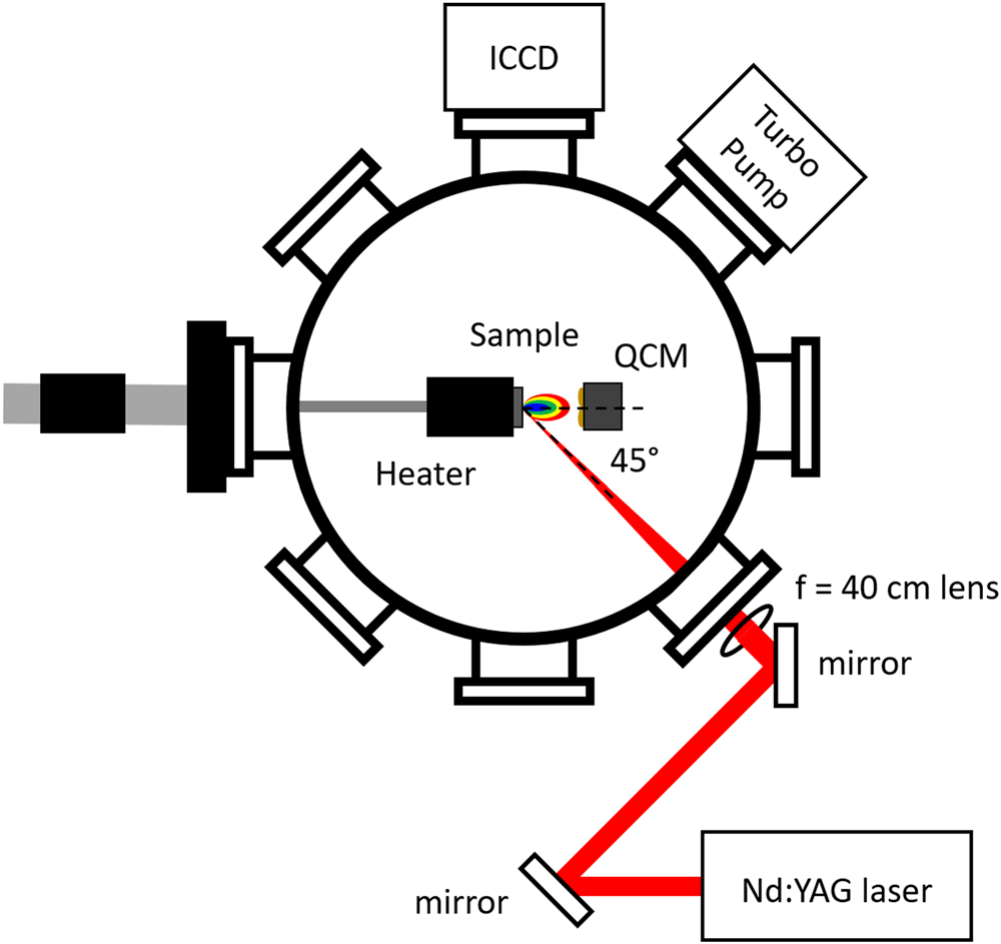
Laser irradiation chamber setup schematic.

**Figure 2:**
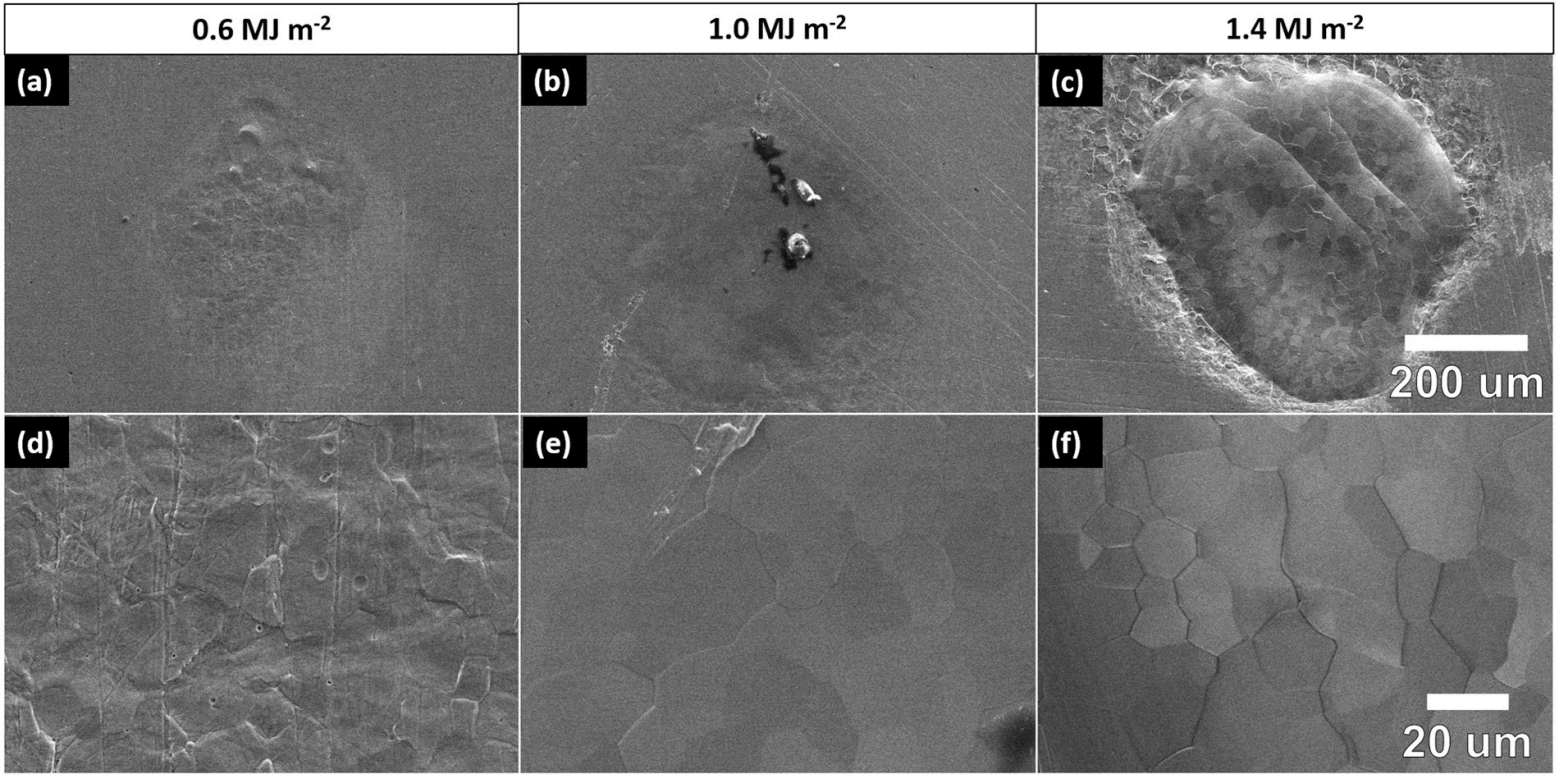
Low-magnification and high-magnification SEM micrographs of Mo surface after 100 shot exposures at (**a**,**e**) 0.6 MJ m^−2^, (**b**,**f**) 1.0 MJ m^−2^, and (**c**,**f**) 1.4 MJ m^−2^.

Energy density dependent testing was done by exposing mirror-finished Mo samples to 100 pulses of 1064 nm Nd:YAG millisecond (ms) laser irradiation at increasing intensities, from 0.6 MJ m^−2^ to 1.8 MJ m^−2^. The ms laser utilized a pulse duration of 1 ms and a repetition rate of 1 Hz, to match expected heat loading conditions during an unmitigated type-I ELM event^29^. A schematic of the experimental setup can be found in Fig. 1. Characterization of irradiated Mo samples using SEM revealed how the surface evolves and eventually melts with increasing energy density. Figure 2 shows the change in surface morphology within the exposed 1 mm spot below, at, and above the threshold of surface melting. Below 1.0 MJ m^−2^ (Fig. 2(a) and (d)), the surface structure appeared to be slightly damaged and rough, but not melted. Previous studies have also discovered a very similar microstructure at low intensities on W^41^. Vertical lines in Fig. 2(d) are due to mechanical polishing. At 1.0 MJ m^−2^ (Fig. 2(b) and (e)), the surface appears molten, exhibiting lower surface roughness and recrystallization. Pulsed heat loading at 1.0 MJ m^−2^ was not sufficient to remove surface imperfections within the exposed spot, revealing that the energy density used could be close to the surface melting threshold. Further increasing the energy density to 1.4 MJ m^−2^ (Fig. 2(c) and (f)) led to higher levels of melting, with the appearance of multiple molten layers, delineated by ripples within the irradiated area. Repeated loading of the surface above the melting threshold led to the formation of multiple molten layers that propagated outward. Recrystallized grains appeared to be of similar size to those seen after pulsed heat loading at 1.0 MJ m^−2^ (Fig. 2(e)), which contrast with the grain growth observed on W^42^. Potential differences in grain growth between materials need to be investigated further with materials of similar starting microstructures. The periphery of the exposed spot in Fig.  2(c) exhibited high levels of surface roughening.

**Figure 3:**
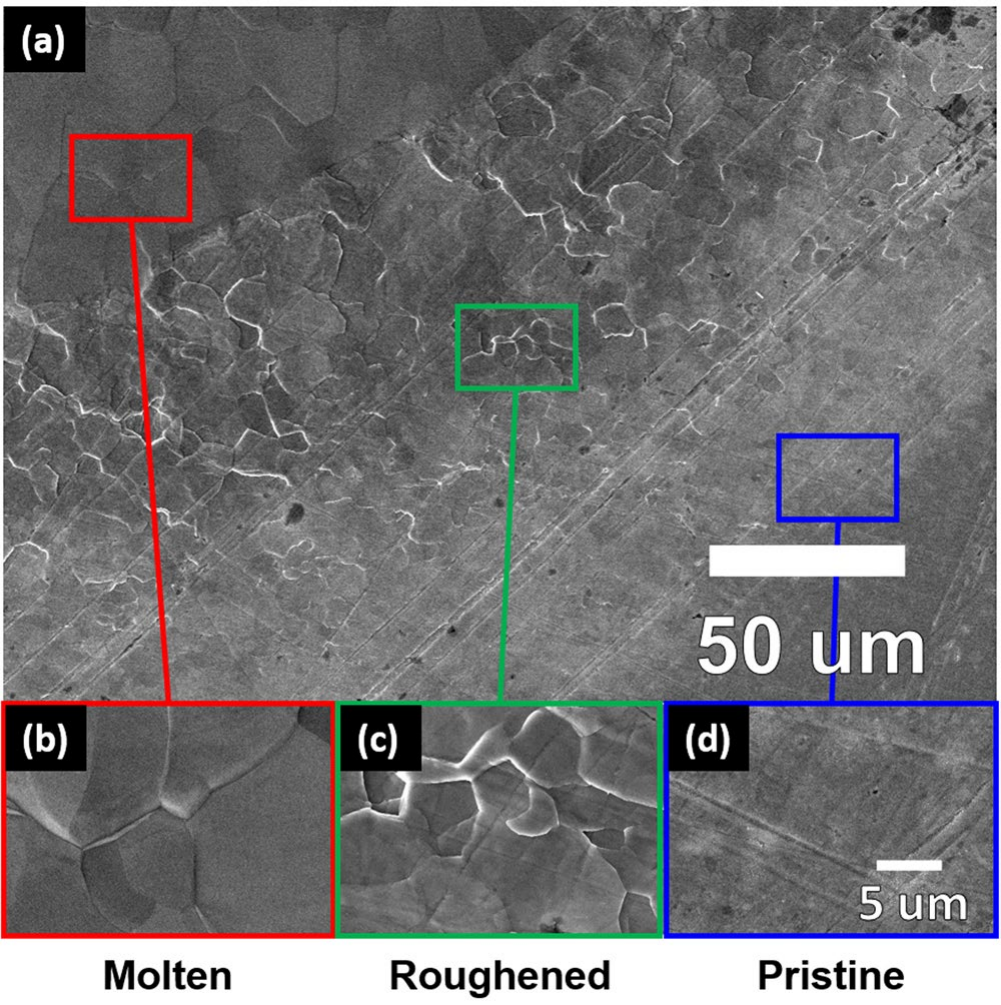
SEM micrographs of three-region morphology that is representative of heat loading above the melting threshold. High-magnification insets shown in parts (**b**,**c**,**d**) reveal the characteristic morphology in each region.

SEM micrographs in Fig. 3 further illustrate the three-region morphology observed due to pulsed heat loading above the melting threshold. The center of each exposed spot (Fig. 3(b)) exhibited complete surface melting and melt motion that propagated radially outward. Mo grains were clearly visible within the molten region – most likely a result of recrystallization after laser irradiation. A roughened region (Fig. 3(c)) was seen along the periphery of the molten region. Laser-produced thermal excursions led to local annealing and deformation of the Mo surface without exceeding the bulk melting point. A similar surface morphology was observed in the center region of spots exposed to pulsed heat loading below the melting threshold. However, the ms laser used for pulsed heat loading utilizes a flat top beam mode, which means that the energy density should have been constant over the entire exposed area. Therefore, the presence of annealing and roughening on the periphery of the molten, laser-exposed spot indicated that heat was being transferred in the radial direction, outward from the center. The outer most region was the pristine surface (Fig. 3(d)) that was not heated significantly by the beam. A three-region surface morphology has also been observed in other heat loading studies on W, with varying spatial profiles^43^. The presence of ripples within the center region reflects the outward motion of molten material during pulsed heat loading. However, SEM analysis cannot fully characterize the dimensions and periodicity of the molten layers.

**Figure 4:**
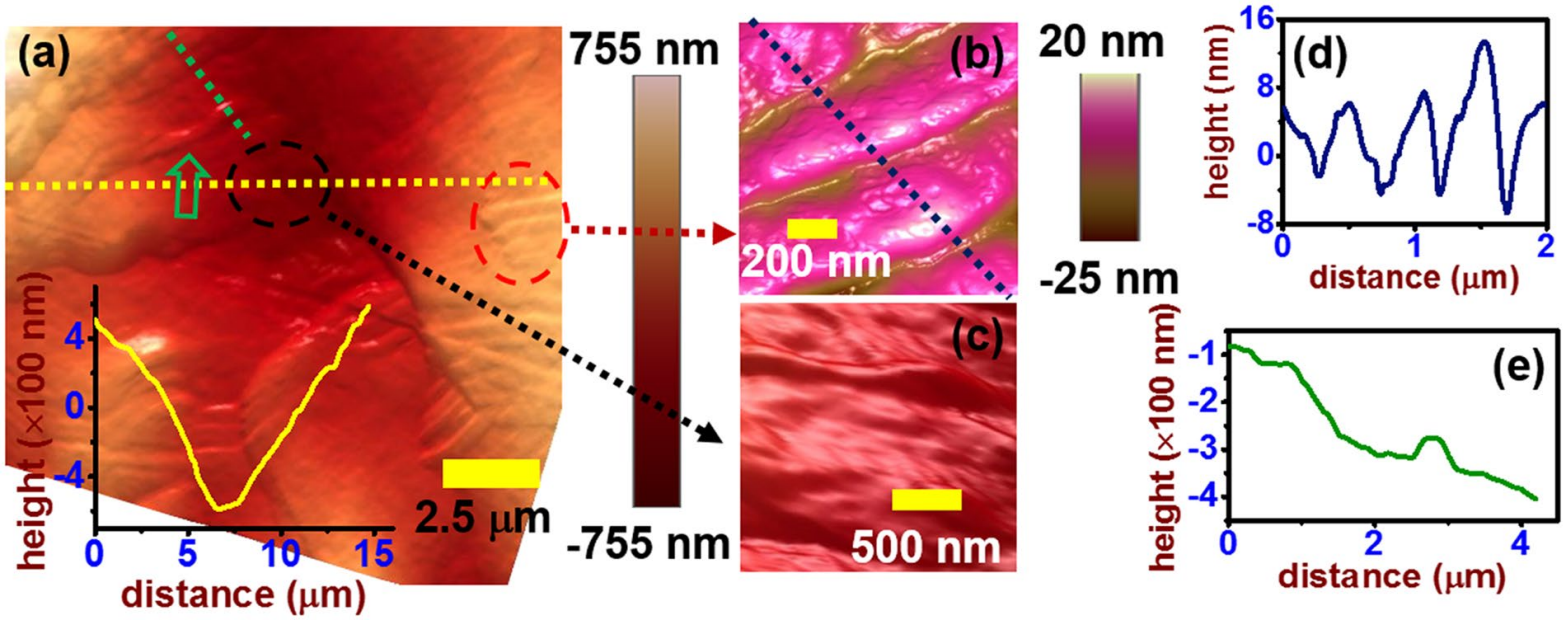
High-resolution atomic force microscopy (AFM) 3D topography mode images from Mo exposed to 100 shots at 1.6 MJ m^−2^.

Further analysis of melt layer characteristics using AFM on a spot exposed to 100 laser pulses at 1.6 MJ m^−2^ provided insight into the movement and topography of molten layers. Figure 4 shows the typical topographical mode 3D AFM image from the molten region. The inset of Fig. 4(a) shows the line profile along the dotted yellow line. The scanned area reveals a clear peak and valley structure, which is due to the stratification of different molten layers. A molten ripple structure is further observed in higher magnification images in Fig. 4(b) (taken within dotted red circle denoted in Fig. 4(a)). This structure has a periodicity of ~500 nm and an average height of ~12 nm (as shown in Fig. 4(d)). Another example of the ripple structure is shown in Fig. 4(c), taken from the area denoted by the dotted black circle in Fig. 4(a). Figure 4(e) represents the line profile of the surface on a smaller scale than that shown in the inset of Fig. 4(a). The line profile was measured along the dotted green line shown in Fig. 4(a). The line profile further confirms the presence of a stratified morphology. The bump in Fig. 4(e), at a distance of ~2.8 µm, clearly shows the ridge of one molten layer (marked in Fig. 4(a) with green arrow). The FWHM of the bump is ~300 nm and height is ~60 nm. Evidence of stratified layers along the Mo surface further supports the presence of melting.

### Particle emission and surface excitation

Events of high heat loading can not only damage and possibly melt the PFC surface, but also introduce PFC material into the fusion plasma. Such contamination needs to be avoided during operation to ensure high plasma performance. High-Z materials, such as W and Mo, possess very low impurity thresholds due to their high atomic number^44^. Past studies have found that a W atomic concentration higher than 10^−5^ could lead to quenching of the core plasma^45–47^. While contamination is not a concern during steady-state operation, transient events in the plasma, such as type-I ELMs, will impart very high heat fluxes on the PFC surface that could lead to melting and splashing of the material surface. As a result, molten droplets could leave the surface and enter the plasma. The current experiment attempted to introduce some novel methods for detecting and predicting material loss. Imaging of the plasma plume emitted after irradiation was done using an ICCD camera (refer to Fig. 1 for alignment). The device was used to correlate photon excitation by emitted species to the material response during pulsed heat loading. The intensity of emission provided a qualitative metric on the degree of heating of the Mo surface due to laser irradiation. In addition, the size and shape of the detected plume was representative of the surface evolution from heat loading.

**Figure 5:**
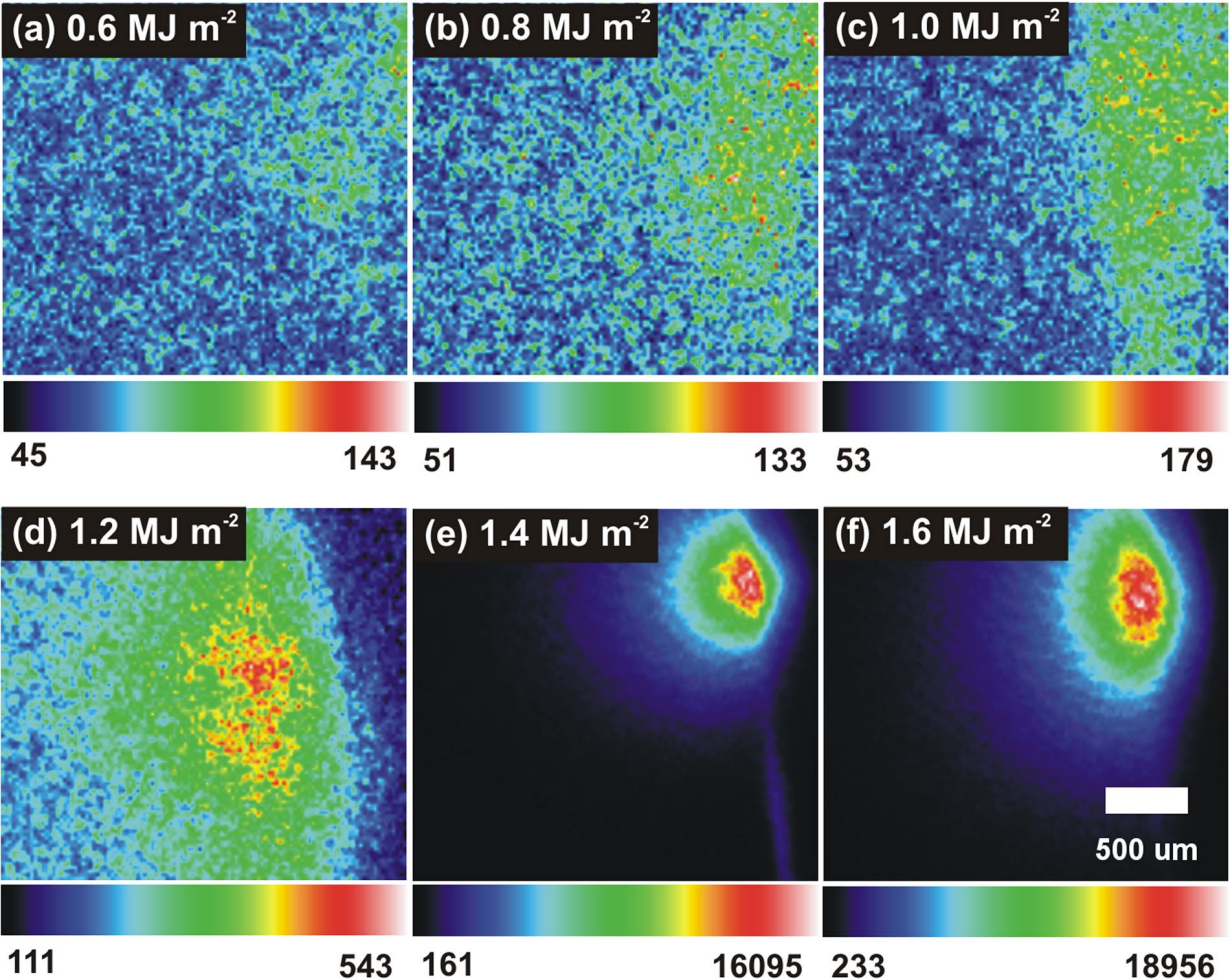
ICCD images of the plasma plume emitted from the Mo surface in response to heat loading after 100 shots at different energy densities. The color of each pixel in an image corresponds to a certain photon intensity. Each image has its own intensity scale.

During each exposure, ICCD images were taken at different shot counts. Figure 5 shows the evolution of the plasma plume as a function of energy density after 100 shots. The color of each pixel corresponds to a certain intensity, and each image is scaled to itself. The intensity of each photon is directly related to the level of excitation experienced by the Mo lattice during laser irradiation. The minimum and maximum intensity levels are displayed underneath each image. Below 1.0 MJ m^−2^, plume emission appeared to be very minimal, which correlates well with imaging of the surface. At 1.2 MJ m^−2^, a moderate increase in the maximum intensity could be attributed to the formation of a molten layer above 1.0 MJ m^−2^, as seen in Fig. 2. At the melting point, particles are more energetic and more mobile. Local hot spots could also lead to partial evaporation of the surface, which would increase the plume excitation. Continuing to increase the energy density led to a drastic increase in the intensity of energetic photons. At 1.4 MJ m^−2^, the maximum intensity climbed two orders of magnitude, indicating a significant change in the response of the Mo surface. Images of the plasma plume at 1.6 MJ m^−2^ reveal enormous photon intensities relative to those found below 1.4 MJ m^−2^. SEM micrographs reveal that the large increase in photon excitation was due to the growth and propagation of the melt layer. Incident, energetic photons transfer heat away from the center of the spot, increasing the amount of molten Mo. The now-mobile material begins to move and splash, resulting in a plasma plume that consists of more energetic photons due to increased levels of ejected material. Continued heating of the melt layer will lead to evaporation, despite its high specific heat of vaporization^30^. Images of the plume clearly show that the energy density has a significant impact on the excitation of emitted species.

**Figure 6:**
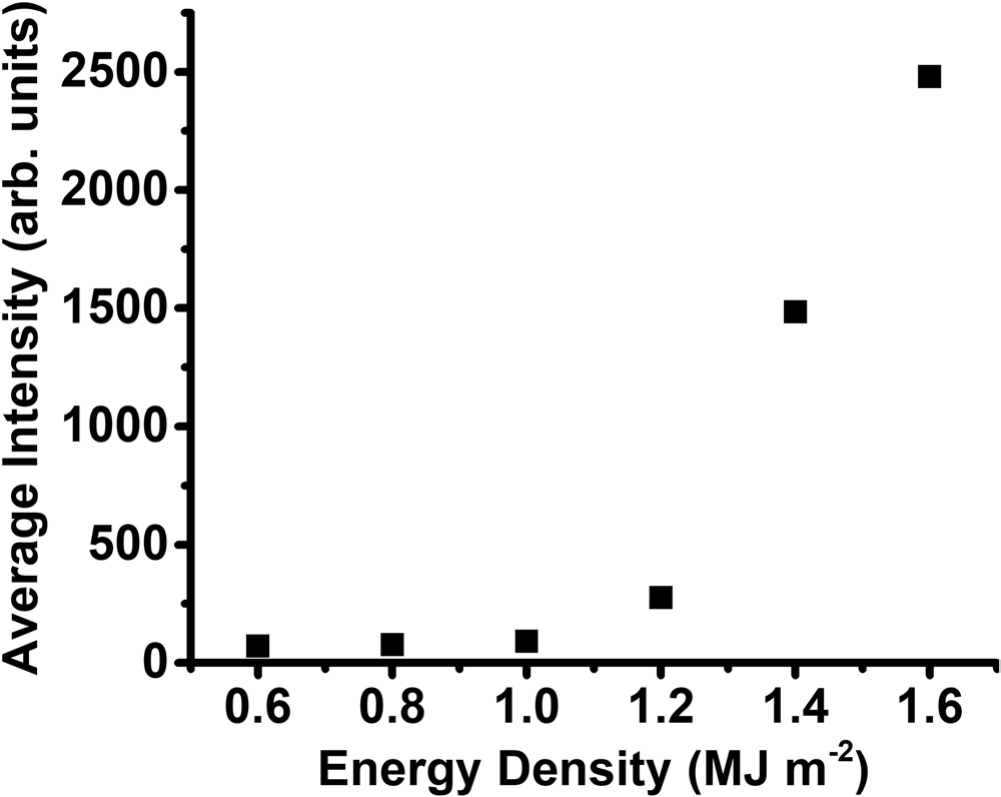
Average intensity of photon emission at increasing energy densities. Each value was calculated from the ICCD image taken after the 100^th^ laser shot.

Finding the average intensity of emitted photons from the ICCD images helps in defining the relationship between energy density and material loss during heat loading. The average photon intensity after 100 shots is plotted as a function of energy density in Fig. 6. The data further bolsters the observations made on the images in Fig. 5; intensity remains very low until the laser energy density surpasses ~1.2 MJ m^−2^. The distinct change in excitation correlates well with previous findings that surface melting occurs on W at ~1.4 MJ m^−2^ ^24^. Although laboratory experiments cannot predict exact behavior in a reactor environment due to several constraints, clear trends in photon excitation suggest that safe operating windows can be specifically defined and controlled to avoid possible contamination of the core plasma.

Multiple studies have been conducted to help characterize the evolving material response during ELM-like heat loading at higher intensities. Work presented in^48^ effectively theorizes how the PFC surface responds to transient pulses of increasing magnitude. Near the melting threshold, pulsed heat loading will create a small melt layer that will recrystallize once the pulse terminates^48^. Then, as the energy density continues to increase, the molten layer will move away from the center, creating a ripple-like structure on the periphery of the exposed spot. Finally, at high enough energy densities, the intense heat from the transient pulse begins to cause evaporation and droplet formation. The existence of a melting threshold and an emission threshold can be inferred from the results obtained in the current study via SEM, AFM, and ICCD imaging. Figure 6 clearly shows that at 1.2 MJ m^−2^, the plume intensity began to increase as the surface started to melt. SEM images in Fig. 2 also reveal evidence of melting above 1.0 MJ m^−2^. At 1.4 MJ m^−2^, both the surface morphology and the photon excitation changed drastically, as evidenced in both Figs 2 and 6. The large increase in excitation at 1.4 MJ m^−2^ was likely due to the presence of droplet ejection and vaporization from the Mo surface^48^. Although exact threshold values will vary depending on the shape of the pulse and the number of pulses, the values provided here demonstrate the mechanisms involved in surface damage during pulsed heat loading as the ELM intensity increases.

Further refining the melting and emission behavior of Mo necessitates the direct measurement of material loss during heat loading using a QCM. Recent attempts to experimentally measure mass removal have utilized *ex situ* methods (e.g. microbalances)^49,50^. However, the mass loss per pulse can sometimes be on the order of nanograms^49^. Possible oxidation from an ambient environment could lead to inaccurate data^27^. A QCM presents an *in situ* alternative to measure the mass loss per pulse. Results obtained in^29^ demonstrated the viability of the QCM as a possible tool to measure material loss, yielding consistent results with SEM imaging and optical reflectivity measurements. Work done in^51^ further discuss the use of a QCM as a mass sensor.

One important factor regarding QCM measurements is that data does not represent the entire amount of mass removed from the surface. Previous work studying laser ablation of different materials show that ejected particles are not emitted isotropically^52,53^. With the QCM placed normal to the surface plane, at a distance of 20 mm from the surface, only a small fraction of the total ejected material deposits on the surface of the QCM crystal (refer to Fig. 1 for alignment). Nevertheless, the measurements made can provide valuable information on how emission changes with energy density.

**Figure 7:**
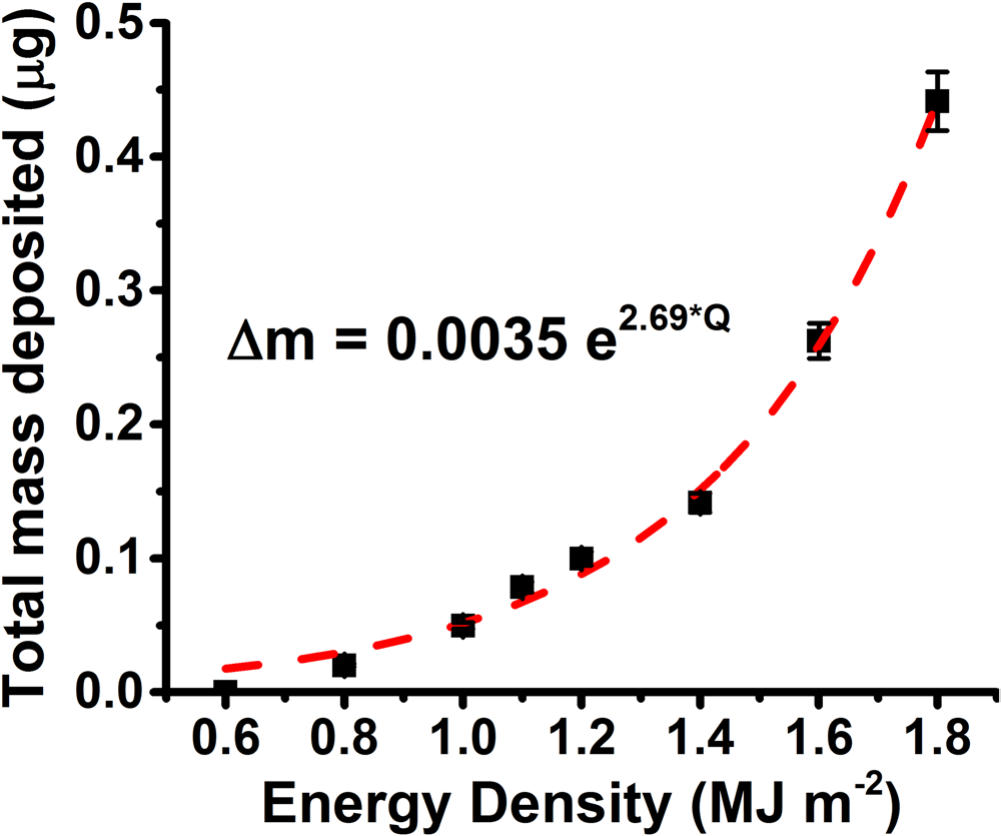
Total mass deposited onto the QCM after 100 shot exposures at varying energy densities with an exponential fit of the form *y* = *ae* ^*bx*^.

The total amount of mass deposited onto the QCM surface after 100 laser shots is shown as a function of absorbed laser energy density in Fig. 7. As the energy density of the laser increased, the amount of mass detected by the QCM appeared to increase exponentially. The nonlinear increase in mass loss is supported by SEM and ICCD images discussed earlier (see Figs 2 and 5). Below 1.0 MJ m^−2^, the mass deposited onto the QCM was almost negligible (<50 ng), which was likely due to a combination of noise and potential emission of surface oxides. Between 1.0 MJ m^−2^ and 1.4 MJ m^−2^, emission began to increase due to the onset of surface melting (as observed in Fig. 2). The growth of the melt layer leads to higher levels of emission via splashing and possible droplet formation. Vaporization of the surface will also contribute to mass loss above a certain intensity.

Exponential increases in mass loss with energy density has been observed in other work done on W. Studies presented in^49^ found that particle emission increased exponentially as power density increased. Exposures were done using a plasma accelerator to replicate ELM-like heat loading. Using an *ex situ* microbalance for mass loss measurements, emission appeared to follow the equation

1 $${\rm{\Delta }}m(Q)=0.02\,exp(3.5Q)$$

where $\triangle m$ is the specific mass loss and Q is the energy density^49^. The first term, 0.02, will be referred to as the scaling term. The second term, 3.5, will be referred to as the growth term. Although the absolute values of emission obtained in the current study are not directly comparable with those obtained in the previous study, because experimental parameters and conditions were different, the rate of mass loss is comparable. Comparing the emission behavior of W and Mo under similar irradiation conditions would provide a crude indication of which material performs better under ELM-like heat loading.

Based on the analysis done in^49^, an exponential function of the same form was fit to the data in Fig. 7. The exponential function fits the data well, with a coefficient of determination (R^2^) of 0.994 (Fig. 7). A high coefficient of determination indicates that the fit can be used to accurately predict future behavior. The exponential curve fit yields the equation

2 $${\rm{\Delta }}m(Q)=0.0035\,exp(2.69Q)$$

with a growth term of 2.69. Possible reasons why the growth term for Mo is less than that for W could be due to the heterogeneity in particle emission or to the higher specific heat of vaporization of Mo. The values obtained do not provide a definitive answer on which material results in less mass loss, but instead illustrate that both materials undergo mass loss that increases exponentially with increasing ELM intensity. Future work should conduct direct comparisons between candidate PFC materials to determine which material possesses a lower growth term, and therefore causes less contamination during an ELM event. Recent work has shown that mass loss also behaves exponentially in the presence of helium-induced “fuzz formation”^54^. Additional diagnostics will be needed to define the mechanisms of melt layer splashing and particle emission under different loading conditions. Predicting levels of contamination during type-I ELM events is of utmost importance in minimizing plasma impurities and extending component lifetimes in future devices.

## Conclusion

Pulsed heat loading experiments performed on a Mo surface indicate that the onset of surface melting and droplet ejection at higher ELM intensities (above 1.0 MJ m^−2^) make long-term operation unsustainable. Heat loading replicated by a pulsed Nd:YAG ms laser led to surface melting above 1.0 MJ m^−2^ (32 MJ m^−2^ s^−1/2^) and enhanced particle emission above 1.4 MJ m^−2^ (44 MJ m^−2^ s^−1/2^). The magnitude of these thresholds will vary, based on the number of applied pulses and the shape of the pulse, but remain an important indicator of the evolving surface morphology as a function of ELM intensity^39,40^. Imaging done via SEM illustrates the change in surface morphology as energy density increases. Above 1.0 MJ m^−2^, the surface begins to show signs of recrystallization. Above 1.4 MJ m^−2^, radial propagation of molten layers away from the center of the laser-exposed area reveal the occurrence of melt motion and potential splashing. Topographical analysis done using AFM confirms the presence of molten ripple morphology within melted spots. Motion of the molten layer in a reactor environment will also be affected by the J × B force. Future experiments that incorporate magnetic field effects would therefore provide valuable insight into how melt motion depends on ELM parameters and irregular surface morphology (e.g. fuzz formation). Material ejection was studied directly via QCM measurements and indirectly via ICCD imaging. Above 1.2 MJ m^−2^, photon excitation of the plasma plume increased by two orders of magnitude, indicating the presence of molten or vaporized material. *In situ* mass loss measurements correlated well with ICCD data, showing an exponential increase in emission. Inspired by previous work on ELM-induced particle emission from W, the QCM measurements were fit to an exponential empirical formula^49^. The formula fit the data very well, indicating that mass loss could become a serious problem with regards to core plasma contamination without the mitigation of type-I ELMs. QCM analysis did not show an appreciable difference in the emission behavior between W and Mo. Follow-up studies will further investigate the mechanisms behind the exponential increase in mass loss from refractory metals.

Future PFC development needs to continuously examine how different materials and surface structures perform under transient heat loading to ensure acceptable material lifetimes and contamination levels for ITER and future DEMO. The effects of material composition and material working should be studied to optimize the physical and thermal properties of the component. Previous work done on alternative materials has shown that utilizing Ta and Y_2_O_3_ as alloying elements can improve the thermal shock performance of the PFC surface^6,55^. Work should also be done on the synergistic effects between transient heat loading and other damage processes that take place in a fusion environment. These processes include tritium retention, helium-induced fuzz formation, and neutron irradiation. Recent work has presented conflicting results on whether fuzz formation improves or degrades the thermal response of the PFC surface during ELM-like heat loading^15,29,50,54,56^. Designing experiments that study the surface response of candidate materials after simultaneous particle and heat loading is critical to understanding the severity of transient, off-normal events in next step fusion devices.

## Methods

Heat loading experiments were conducted in the Ultra-High Flux Irradiation (UHFI) laboratory at the Center for Materials Under Extreme Environment (CMUXE) at Purdue University. The samples used in this experiment measured 10 mm × 10 mm, and were cut from a 99.95% purity, Mo foil of 0.5 mm thickness. After being cut, each sample was mechanically polished to a mirror finish. A schematic of the experimental setup used for irradiation experiments is shown in Fig. 1. A 1064 nm pulsed Nd:YAG ms laser was used for this experiment, because of its ability to replicate the intensity and duration of type-I ELMs. The laser utilizes a flat top beam mode, which results in uniform heating over the entire beam area. The laser had a pulse duration of 1 ms and a spot size of ~1 mm.

Heat loading studies were performed by varying the intensity of laser exposure. As stated previously, unmitigated type-I ELMs possess energy densities of about 0.2–2.0 MJ m^−2^, with a duration of 0.1–1 ms^29^. Energy density dependent studies consisted of 100 shot exposures, with intensities varying between 0.6 and 1.8 MJ m^−2^. Preliminary analysis of these exposures found that significant surface melting began at an intensity of ~1.2 MJ m^−2^.

During irradiation, a QCM was used *in situ* to detect particle emission from the sample surface. The QCM crystal was placed normal to the sample surface at a distance of ~20 mm (Fig. 1). Previous experiments on the angular dependence of emission show that particles leave the surface with a bias normal to the surface regardless of small variations in the incident angle^52,53^. Before every exposure, the QCM was zeroed. A time-integrated value from that exposure was then recorded.

Plume expansion was also studied by using an intensified charge-coupled device (ICCD) camera to image particle excitation. Images were taken after the 100^th^ laser shot at varying energy densities. During irradiation, the camera focused on the sample surface and the area in front of the sample.

Surface characterization was conducted after exposure via *ex situ* field emission (FE) scanning electron microscopy (SEM). Imaging helped qualitatively characterize the Mo surface after pulsed heat loading and track the evolution of the molten layer above the melting threshold. In addition, atomic force microscopy (AFM) was performed to measure the height profile and general topography of the molten layers for different samples that exhibited melting. By combining emission detection methods and surface morphology characterization, a comprehensive analysis of the heat loading process on Mo was developed.

### Data Availability

The datasets generated during and/or analyzed during the current study are available from the corresponding author on reasonable request.
